# Nocturnal Birds of Prey as Carriers of *Staphylococcus aureus* and Other Staphylococci: Diversity, Antimicrobial Resistance and Clonal Lineages

**DOI:** 10.3390/antibiotics11020240

**Published:** 2022-02-12

**Authors:** Vanessa Silva, Ana Filipa Lopes, Vanessa Soeiro, Manuela Caniça, Vera Manageiro, José Eduardo Pereira, Luís Maltez, José Luis Capelo, Gilberto Igrejas, Patrícia Poeta

**Affiliations:** 1 Microbiology and Antibiotic Resistance Team (MicroART), Department of Veterinary Sciences, University of Trás-os-Montes and Alto Douro (UTAD), 5000-801 Vila Real, Portugal; vanessasilva@utad.pt (V.S.); jeduardo@utad.pt (J.E.P.); lmaltez@utad.pt (L.M.); 2 Department of Genetics and Biotechnology, University of Trás-os-Montes and Alto Douro (UTAD), 5000-801 Vila Real, Portugal; gigrejas@utad.pt; 3 Functional Genomics and Proteomics Unit, University of Trás-os-Montes and Alto Douro (UTAD), 5000-801 Vila Real, Portugal; 4 LAQV-REQUIMTE, Department of Chemistry, NOVA School of Science and Technology, Universidade Nova de Lisboa, 2829-516 Caparica, Portugal; 5 Wildlife Study and Rehabilitation Centre (CERAS), Quercus ANCN, Rua Tenente Valadim, 6000-284 Castelo Branco, Portugal; anafilipa.sl@gmail.com; 6 Wildlife Rehabilitation Centre of Parque Biológico de Gaia, Rua da Cunha, 4430-812 Avintes, Portugal; vanessasoeiro@cm-gaia.pt; 7 National Reference Laboratory of Antibiotic Resistances and Healthcare Associated Infections (NRL-AMR/HAI), Department of Infectious Diseases, National Institute of Health Dr. Ricardo Jorge, Av. Padre Cruz, 1649-016 Lisbon, Portugal; manuela.canica@insa.min-saude.pt (M.C.); vera.manageiro@insa.min-saude.pt (V.M.); 8 Centre for the Studies of Animal Science, Institute of Agrarian and Agri-Food Sciences and Technologies, Oporto University, 4051-401 Oporto, Portugal; 9 Associate Laboratory for Animal and Veterinary Science (AL4AnimalS), Veterinary and Animal Research Centre, University of Trás-os-Montes and Alto Douro (UTAD), 5000-801 Vila Real, Portugal; 10 BIOSCOPE Group, LAQV@REQUIMTE, Chemistry Department, Faculty of Science and Technology, NOVA University of Lisbon, 2825-466 Almada, Portugal; jlcm@fct.unl.pt; 11 Proteomass Scientific Society, Costa de Caparica, 2825-466 Almada, Portugal

**Keywords:** *Staphylococcus aureus*, MRSA, *mec*C, ST1245-t843, antimicrobial resistance, CoNS

## Abstract

Owls are nocturnal predators that inhabit urbanized and farmlands. They are in direct contact with other animals, both livestock and small wild rodents that they mostly feed on. Staphylococci can be both commensal and pathogenic bacteria that are widespread across the various ecological niches. We aimed to isolate staphylococci from owls and to characterize their antimicrobial resistance, virulence factors and genetic lineages. Swab samples were collected from the throat and cloaca of 114 owls admitted to two rehabilitation centers in Portugal. The identification of staphylococci species was performed by MALDI-TOF. Staphylococci antimicrobial resistance and virulence genes were investigated by means of the disk diffusion method and PCR. *Staphylococcus aureus* isolates were characterized by MLST, *agr* and *spa*-typing. Of the tested animals, 66 isolates were recovered, including 10 different species of staphylococci, of which 25 were coagulase-positive (CoPS) and 41 were coagulase-negative (CoNS). Twenty-three *S. aureus* were isolated, of which one *mec*C-MRSA was identified. The isolates were mainly resistant to penicillin, aminoglycosides, clindamycin and tetracycline. *mec*C-MRSA belonged to ST1245 and *spa*-type t843 and the remaining *S. aureus* were ascribed to 12 STs and 15 *spa* types. A high diversity of clonal lineages was identified among the *S. aureus* isolated from wild owls. Owls feed mainly on small rodents often exposed to waste and anthropogenic sources, which may explain the moderate prevalence of *S. aureus* in these animals.

## 1. Introduction

*Staphylococcus* spp. are abundant colonizers of the normal microflora of humans and animals [1]. Despite living in commensalism with the host, staphylococci, in particular, *Staphylococcus aureus*, can cause a wide spectrum of infections [2]. The *Staphylococcus* genus comprises the coagulase-negative staphylococci (CoNS) and coagulase-positive staphylococci (CoPS) [1]. CoNS have fewer virulence factors than *S. aureus* and were generally considered contaminants rather than pathogens [3,4]. Nevertheless, recent studies have shown that CoNS have an increasing clinical impact and can act as opportunistic pathogens, particularly in immunocompromised patients [1,4,5,6]. Staphylococci can easily acquire antimicrobial resistance genes, preventing the treatment of some infections [2]. Over the last few decades, methicillin-resistant *S. aureus* (MRSA) have been a leading cause of nosocomial infections and an emergent zoonotic pathogen [7]. Methicillin resistance in staphylococci is generally promoted by the *mec*A or *mec*C, including several allotypes, genes which encode for a penicillin-binding protein (PBP2a) that has a low affinity for β-lactam antimicrobials [8,9]. These genes are located on a mobile genetic element called the Staphylococcal chromosome cassette *mec* (SCC*mec*). SCC*mec* elements are highly diverse and are currently classified into 14 types as well as various subtypes [10]. The *mec*C gene was first reported over a decade ago and since then it has been detected in staphylococci isolated from several different hots and sources [11,12,13,14]. More recently, *mec*D and plasmid-borne *mec*B genes have also been identified in *S. aureus* and Macrococcus caseolyticus, respectively [9,15]. Staphylococci, both methicillin-resistant and -susceptible, have been found among a taxonomically diverse range of animals including mammals, reptiles, fish, crustaceans and birds [16,17,18,19,20]. While the prevalence, antimicrobial resistance and clonal lineages of *S. aureus* and CoNS from livestock and companion animals was subject of intensive research, studies on strains isolated from the environment and wild animals are scarcer [7,21].

Routes of transmission of antimicrobial-resistant bacteria (ARB) between humans, farm animals, pets and wild animals are not fully understood. Wild birds, with their capacity for long-range movements, can carry ARB over long distances and contribute to the dissemination of those bacteria [22]. Environmental contamination of wild bird feces may reach surface waters, agricultural fields, livestock and companion animals, and locations with anthropogenic activity, increasing the risk of bacterial transmission [23,24]. Wild birds can carry a wide range of different multidrug-resistant bacteria, including staphylococci [19,25,26]. Livestock farms and landfills are a potential source of staphylococci detected in these animals. Furthermore, predatory birds can also feed on carcasses and small animals which, in turn, may also by carriers of ARB [27]. Owls are nocturnal predators that regularly inhabit woodlands and farmlands but also inhabit habitats that are urbanized due to their adaptation to anthropogenic environments [28,29]. These owls established in the Mediterranean region due to favorable climatic conditions [21]. In Portugal, the most common owl species are: Barn (*Tyto alba*), Tawny (*Strix aluco*) and Little (*Athene noctua*) [30]. These particular species are also widely distributed in the central and northern Eurasia subcontinent and north Africa [31,32,33]. Owls feed mainly on small mammals, birds, amphibians and a wide range of invertebrates, including wild mice and harmful insects [31,34]. Therefore, to better understand the molecular epidemiology of *S. aureus* and the frequency of colonization and antimicrobial resistance of CoNS in nocturnal predatory birds, we isolated staphylococci from owls admitted to two rehabilitation centers in Portugal and characterized the isolates regarding antimicrobial resistance, virulence factors and genetic lineages.

## 2. Results

### 2.1. Frequency and Distribution of Staphylococci in Night Prey

In this study, swab samples were collected from 114 owls. A total of 54 (47.4%) owls carried staphylococci, of which 9 carried more than one *Staphylococcus* species. Co-carriage of two different species was identified in seven owls, and four species in one animal. From the 43 tawny owls (*Strix aluco*), 41 barn owls (*Tyto alba*), 25 little owls (*Athene noctua*) and 5 Eurasian eagle-owls (*Bubo bubo*) sampled, 25 (58.1%), 13 (31.7%), 13 (52%) and 3 (60%) were positive for staphylococci, respectively (Table S1). Regarding the isolates, 66 were recovered including 10 different species of staphylococci, of which 25 were coagulase-positive (CoPS) and 41 were coagulase-negative (CoNS). From the 25 CoPS, 23 were identified as *S. aureus* and the remaining two were *Staphylococcus pseudintermedius*. CoNS included 22 *Staphylococcus sciuri*, 11 *Staphylococcus lentus*, 2 *Staphylococcus vitulinus*, 2 *Staphylococcus haemolyticus*, 2 *Staphylococcus xylosus*, one *Staphylococcus saprophyticus* and one *Staphylococcus succinus*. The staphylococci distribution among the four owl species in shown in Table 1. *S. epidermidis* and *S. xylosus* were isolated only from *Athene noctua* and *Strix aluco*, respectively, while *S. aureus* and *S. sciuri* were present in all four owl species.

### 2.2. Characterization of CoPS Isolates

All CoPS were characterized regarding the presence of antimicrobial resistance and virulence genes. *S. aureus* isolates were also typed by MLST, *spa*- and *agr*-typing (Table 2). From the 23 *S. aureus* isolates, only one was resistant to cefoxitin and harbored the *mec*C gene. The MRSA isolate was also resistant to penicillin and carried the *bla*Z-SCC*mec*XI gene. The following genes encoding virulence factors *hla*, *hlb* and *etd*2 were also detected. The *mec*C-MRSA isolate was ascribed to ST1245, which belonged to the clonal complex (CC) 130, *spa*-type t843 and *agr* type III. From the 22-remaining methicillin-susceptible *S. aureus* (MSSA), 11 (50%) were susceptible to all antibiotics tested. Ten MSSA isolates were resistant to penicillin and six harbored the *bla*Z gene. The *tet*K gene was detected in the two isolates showing resistance to tetracycline. Two isolates had phenotypic resistance to macrolides and lincosamides and carried the *erm*A and *mph*C genes. Regarding the presence of virulence factors, all isolates carried at least one virulence gene, with the *hla* gene being present in all isolates and the *hlb* gene in 15 isolates. Six isolates were positive for the *scn* gene, which is a marker of the Immune Evasion Cluster (IEC) and were further screened for the presence of the *chp*, *sak*, *sea* and *sep* genes to determine the IEC group [35]. Four isolates harbored the *scn*, *sak* and *chp* genes and were ascribed to IEC type B and 2 isolates carried the *scn* and *sak* gene and were assigned to type E. MSSA isolates were ascribed to 13 STs and 15 *spa* types. The isolates were distributed among the four *agr* types. Finally, *S. pseudintermedius* isolates (VS2983 and VS2984) were susceptible to all antibiotics tested but one carried the *mec*A gene.

### 2.3. Characterization of CoNS Isolates

All CoNS were characterized regarding their phenotypic and genotypic antimicrobial resistance (Table 3). Out of the 22 *S. sciuri*, 8 were susceptible to all antibiotics tested. Eight isolates carried the *mec*A gene, which is known to be responsible for methicillin resistance. Resistance to clindamycin and tetracycline was detected in six and two isolates, respectively, conferred by the presence of the *mph*C and *tet*K genes. From the 11 *S. lentus* isolates, 3, 5 and 4 showed resistance to penicillin, clindamycin and tetracycline, respectively. As was also the case with the *S. sciuri* isolates, the genes detected were *mec*A, *mph*C and *tet*K. The two *S. epidermidis* isolates were the only ones among the CoNS that carried the *bla*Z gene. Both isolates had resistance to fusidic acid encoded by the *fus*B gene and one isolate also showed resistance to erythromycin conferred by the *msr*(A/B) and *mph*C genes. Regarding the *S. haemolyticus* isolates, one was susceptible to all antibiotics while the other showed resistance to erythromycin, clindamycin and trimethoprim-sulfamethoxazole. The *S. xylosus* isolates carried the *mph*C, *tet*M and *tetL* genes. Finally, the *S. saprophyticus* and one of the *S. vitulinus* isolates carried the *mec*A gene and the *S. succinus* isolate was susceptible to all antibiotics.

## 3. Discussion

This report represents the largest study of staphylococci recovered from healthy wild nocturnal birds of prey. Wild birds as carriers of antimicrobial-resistant pathogens may be considered as a public health problem in the One Health context. Nevertheless, studies on the microflora of birds of prey are scarce and studies on the prevalence of staphylococci in owls are almost inexistent [36]. Therefore, it is not possible to make a direct comparison of the prevalence of staphylococci obtained in this study with other reports. In our study, we investigated the staphylococci colonization of 114 owls of four different species and obtained a moderate staphylococci prevalence of 47.4%. Other studies conducted with wild birds of prey, some of which included a few owls, obtained similar or higher results [25,37,38]. In a study conducted by Dipineto et al., the pellets of 73 birds of prey, including 13 owls, were screened for the presence of staphylococci. In that study, *Staphylococcus* spp. was detected in 64 out of 73 samples, of which 26 (35.6%) were *S. aureus*, but no MRSA was isolated [38]. In our study, the prevalence of *S. aureus* was lower (20.2%). Another study conducted in Spain with 324 samples of wild birds reported a total of 27 (8%) CoPS isolates, which included only 2 staphylococci species: 15 *S. aureus* and 12 *S. delphini* [39]. In our study, we also obtained two species of CoPS; however, these were *S. aureus* and *S. pseudintermedius*. The rate of carriage of CoNS detected in wild owls in our study (36%) was higher than that detected in a previous study conducted in Portugal in wild hares, which suggests that raptors may be natural reservoirs of CoNS [40]. Two studies conducted in Portugal investigated the presence of CoNS in wild birds of prey, including *Strix aluco* and *Athene noctua* owls, and obtained a prevalence of 37.5% and 75% of CoNS [19,37]. The species isolated from owls were *S. sciuri* (*n* = 3), *S. xylosus* and *S. saprophyticus.* In our study, the most frequent species detected was also S. sciuri (22 out of 41 CoNS), and *S. xylosus* and *S. saprophyticus* were also isolated.

CoPS were detected in 25 (21.9%) wild owls. All four species of owls carried *S. aureus,* but a higher incidence of *S. aureus* (32%) was found in *Athene noctua*. One MRSA strain was isolated from *Athene noctua* and carried the *mec*C gene. Therefore, as far as we know, this is the first study reporting a *mec*C-postive MRSA isolated from owls. In addition to the *mec*C gene, this isolate also harbored *bla*Z-SCC*mec*XI, which is a *bla*Z allotype associated with SCC*mec* XI as previously reported [13]. In turn, the SCCmec XI is also associated with the mecC gene [41]. In addition to the *hla* and *hlb* virulence genes, the *mec*C-MRSA isolate also carried the *etd*2 gene, which is an exfoliative toxin that is a homologue to *etd*. The presence of *etd*2 in *mec*C-MRSA has been reported in human and animal strains of CC130 and may indicate an evolutionary step towards host adaptation [42,43,44]. The MRSA isolate was ST1245, which belongs to CC130 and *spa*-type t843. In Portugal, the *mec*C gene has been reported only in two studies, one conducted in wild rodents and another in surface waters, and the clonal lineages detected in those isolates differ from the one identified in this study [13,20]. *mec*C-MRSA belonging to ST1245 has been reported in bovine samples in the UK and in a bat in Germany associated with *spa*-type t843, as well as in horses from France, but, in this case, was associated with a different *spa*-type [45,46,47]. The *mec*C isolate lacked the IEC system genes, which is in accordance with most studies reporting *mec*C-MRSA and suggests a possible animal origin [13,20,48,49]. In fact, the presence of the IEC type E in *mec*C-positive isolates seems to be associated with ST1945 (CC130) since it has only been reported in those isolates [20,50,51]. As expected, the *mec*C-MRSA isolate was found to belong to *agr* type III, which is always associated with the *mec*C gene and CC130 [52].

**Figure 1:**
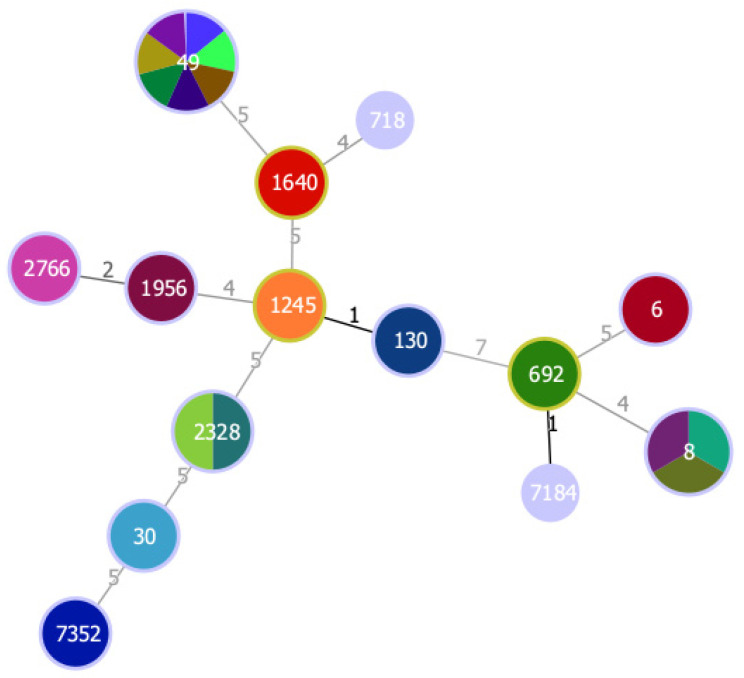
Minimum spanning tree, based on MLST of 23 *S. aureus* isolated from wild owls. The minimum spanning tree graph (MST) was created with PHYLOViZ using the goeBURST algorithm [71]. The dominant STs are represented by the circles with larger diameters. Each color represents one isolate. Numbers on lines indicate locus variants between adjacent nodes.

From the 11 MSSA showing resistance to antimicrobials, only two isolates (VS2973 and VS2978) were multidrug-resistant as they were resistant to three and four classes of antibiotics, respectively. Resistance to penicillin was shown in 12 *S. sciuri* isolates but only 8 carried the *mec*A gene and the *bla*Z gene was not detected, which suggests the presence of other unknown resistance mechanisms or that the breakpoints used for this antibiotic are not precise for CoNS. Two MSSA isolates showed resistance to tetracycline mediated by the *tet*K gene, which encodes efflux proteins [53]. A high diversity of clonal lineages was found among the owl isolates (Figure 1). Seven MSSA isolates belonged to ST49, which were the most frequent ST in this study. ST49 was found among MSSA from *Strix aluco* and *Athene noctua* owls. ST49 was previously reported in voles and mice in Germany, was mostly found to be associated with *spa*-type t208 and *agr* II as in our study, and was also identified as a cause of infection in red squirrels [54,55]. The high frequency of *S. aureus* ST49 in owls may be explained by the owls’ food habits. For instance, *Athene noctua* owls feed mainly on wild mice while *Strix aluco* owls have the ability to hunt for a wide range of prey including rats, mice and synanthropic birds [56]. Furthermore, ST49-t208 *S. aureus* isolates have also been detected in the natural environment in Portugal [13]. One of the ST49 isolates (VS2967) was positive for the *scn* gene and was ascribed to IEC type E. The IEC genes are usually located in Sa3int phages, also known as β-hemolysin-converting phages [57]. The presence of these phages is common in *S. aureus* isolated from humans but is much less frequent in animal isolates [58]. Therefore, the presence of IEC genes in our isolate may suggest a possible human origin. However, *S. aureus* ST49 is extremely rare in humans and has been reported once in a human isolated in 1947 [59]. Three isolates belonged to ST8, *spa*-type t121 and *agr* I, and were assigned to IEC type B. *S. aureus* ST8 is frequently associated with methicillin-resistance in humans and animals [60,61,62,63]. Nevertheless, ST8-MSSA has been isolated from wild goose feces in the USA and it seems common in the natural environment in Portugal since it has been isolated from wild rats and superficial water [13,20,64]. Yet, the ST8-MSSA isolates recovered from the natural environment in Portugal had different *spa* types. The *spa*-type t121 identified in all ST8 isolates of our study seems to be linked with MRSA-ST8 that is frequently isolated in the African continent, where ST8-MRSA belonging to t121 is the most common clone [61]. Two isolates from *Tyto alba* belonged to ST2328, t3750 and *agr* III. This ST2328-MSSA-t3750/III clone seems common in wild animals from the Iberian peninsula since it was previously isolated from small mammals [50] and Iberian ibex [65] in Spain, and wild boars in Spain and Portugal [65,66]. Furthermore, ST2328 belongs to CC133, which is a lineage mostly regarded as animal specific [21]. Two MSSA isolates belonging to CC121 (ST2766 and ST1956) and *agr* IV. *S. aureus* ST2766 and ST1956 (associated with *agr* IV) have already been detected in owls’ most common prey; namely, in wood mouse and common vole in Spain [50], in common vole in Germany and the Czech Republic [54] and in field vole from Germany [54]. The only *S. aureus* isolated from *Bubo bubo* (Eurasian eagle-owl) belonged to ST718, which is an uncommon lineage and is often associated with human communities [67]. *S. aureus* ST30 was isolated from a little owl. This lineage is primarily associated with humans, but is also spread among animals, including wild boars, red deer and birds of prey [19,21,51]. One strain isolated from *Tyto alba* owl was ascribed to ST692 (CC692), t1422 and *agr* I. *S. aureus* CC692 was previously isolated from wild birds of prey, such as tawny owls (*Strix aluco*), golden eagles and white-tailed eagles from Sweden, and red kite from Germany [68]. CC692 seems to be a bird-related lineage since it was previously isolated from poultry and pigeons [68]. One *S. aureus* isolate (VS2981), also isolated from a *Tyto alba* owl, belonged to a lineage first described in this study, ST7184, which is a single locus variant of ST692 with a one-point mutation on the *glpF* locus. Finally, one isolate was ST1640 *agr* IV, which has been identified in horses, sheep and red deer [69,70].

*S. sciuri* was the CoNS species most commonly found among owls in this study and it was isolated from all four species of owls investigated. This is not a surprise since this species is the most primitive staphylococci species, has a broad host range and easily adapts to new hosts and environments [13,72]. Colonization of wild animals by *S. sciuri* has been reported, including wild birds [19,25,37,72]. Additionally, Sousa et al. reported the presence of *S. sciuri* isolates in *Strix aluco* and *Athene noctua* owls, mainly associated with resistance to clindamycin and fusidic acid, which is in accordance with our results [37]. It was hypothesized that the *mec*A gene originated from the *S. sciuri* group species, which includes the *S. sciuri*, *S. lentus* and *S. vitulinus* [73]. In our study, only three *S. sciuri* showed phenotypic resistance to cefoxitin but eight isolates were positive for the *mec*A gene. It was shown that although the *mec*A gene is present among *S. sciuri* strains, they may present susceptibility to β-lactams [74]. In fact, the two *S. vitulinus* isolated in this study carried the *mec*A gene. *S. lentus* was the second CoNS most common species among owls and it was mostly detected in *Strix aluco*. As with the other members of the *S. sciuri* group, only one *S. lentus* isolate was resistant to cefoxitin, but four isolates carried the *mec*A gene. *S. lentus* colonizes the skin and mucous membranes of several animal species. Nevertheless, it is typically associated with livestock and their food products [75]. Although owls regularly inhabit urbanized areas, many inhabit wood and farmlands where they may come into close contact with wild animals and livestock and be colonized by staphylococcus species such as *S. lentus* [29]. Two *S. epidermidis* were isolated in this study, both carrying antimicrobial resistance genes. Interestingly, *S. epidermidis* isolates were only recovered from *Bubo bubo* owls, which are considered the largest nocturnal raptor in Europe (Table S1) [75]. Unlike some owl species, such as *Athene noctua*, which only feed on small animals and insects, *Bubo bubo* feed on a larger variety of animals, including medium prey, for example, hedgehogs, rabbits, partridges and pigeons [76]. In contrast to the other CoNS species, *S. epidermidis* harbored the *bla*Z gene. Both isolates also presented the *fus*B gene, which confers resistance to fusidic acid and is carried on plasmids or a genomic island on the chromosome [76]. Two *S. haemolyticus* were isolated, with one of them being susceptible to all antibiotics. The other *S. haemolyticus* was also isolated from a *Bubo bubo*. This isolate had a multidrug-resistant profile and carried the *mph*C, *msr*(A/B) and *tet*O genes. *S. haemolyticus* has been isolated from wild birds in Brazil [77] and in wild pheasant meat in the Slovak Republic [78]. One isolate of *S. saprophyticus* also harbored the *mec*A gene but was susceptible to all antibiotics tested. *S. saprophyticus* was isolated in one owl in Portugal but it was associated with a multidrug-resistance phenotype [19]. Both *S. epidermidis* and *S. haemolyticus,* followed by *S. saprophyticus,* are the most significant species of CoNS in human infections and the fact that they are widespread among wildlife carrying multiple resistances may lead to increased public health problems [74].

## 4. Materials and Methods

### 4.1. Samples and Bacterial Isolates

From 2018 to 2021, 114 samples were collected from owls admitted to the Wildlife Study and Rehabilitation Centre (CERAS) located in Castelo Branco (central Portugal) and the Wildlife Rehabilitation Centre of Parque Biológico de Gaia (North of Portugal). Swab samples were collected from the throat and cloaca of each animal and were then placed in Stuart’s transport medium and sent to the Medical Microbiology laboratory at the University of Trás-os-Montes and Alto Douro. The swabs were placed in tubes containing Brain Heart Infusion (BHI) broth with 6.5% NaCl and incubated at 37 °C for 24 h. Then, the inoculum was seeded onto Mannitol Salt agar and CHROMagar^TM^ MRSA agar plates and incubated at 37 °C for 24 to 48 h. Up to 4 colonies per plate, showing different colony morphologies, were selected. Isolates’ species were identified by matrix-assisted laser desorption/ionization time-of flight (MALDI-TOF).

### 4.2. Antimicrobial Susceptibility

Antimicrobial susceptibility was tested using the disk-diffusion method against the following antimicrobial agents (in µg/disk): penicillin G (1U), cefoxitin (30), chloramphenicol (30), ciprofloxacin (5), clindamycin (2), erythromycin (15), fusidic acid (10), gentamicin (10), kanamycin (30), linezolid (10), mupirocin (200), tetracycline (30), tobramycin (10) and trimethoprim/sulfamethoxazole (1.25/23.75). The European Committee on Antimicrobial Susceptibility Testing (EUCAST) 2019 guidelines were followed, except for testing with kanamycin, which followed the Clinical and Laboratory Standards Institute (CLSI) 2017 standards [79,80]. The reference strain *S. aureus* ATCC25923 was used as a quality control strain.

### 4.3. Detection of Antimicrobial Resistance and Virulence Genes

All isolates were screened for antimicrobial resistance and virulence determinants by PCR amplification using previously described primers [81]. Isolates were screened for the presence of determinants conferring resistance to: beta-lactams (*bla*Z, *bla*Z-SCC*mec*XI, *mec*A and *mec*C), macrolides and lincosamides (*erm*A, *erm*B, *erm*C, *erm*T, *msr*(A/B), *lnu*A, *lnu*B, *vga*A, *vga*B, *vga*C), tetracycline (*tet*M, *tet*K, *tet*L and *tet*O), aminoglycosides (*aac*(6′)-Ie-*aph*(2′’)-Ia, *ant*(4′)-Ia and *aph*(3′)-IIIa), phenicols (*cat*_pC194_, *cat*_pC221_, *cat_p_*_C223_, *fex*A, and *fex*B), oxazolidinones (*cfr*), trimethoprim/sulfamethoxazole (*dfr*A, *dfr*G, *dfr*K and *dfr*D) and fusidic acid (*fus*B, *fus*C and *fus*D). The virulence genes tested encoded for hemolysins (*hla*, *hlb* and *hld*), Panton-Valentine leuocidin (PVL) (*lukF*/*lukS*-PV), exfoliatins (*eta*, *etb* and *etd*2) and toxic shock syndrome toxin (*tst*) [81]. Additionally, the presence of the *scn* gene, which is a marker of the Immune Evasion Cluster (IEC), was also investigated by PCR. Isolates that were positive for *scn* were further screened for the presence of the *chp*, *sak*, *sea* and *sep* genes to determine the IEC group [35]. Positive and negative controls used in all experiments belonged to the strain collection of the University of Trás-os-Montes and Alto Douro.

### 4.4. Molecular Typing

All *S. aureus* isolates were typed by multilocus sequence typing (MLST), *spa*- and *agr*-typing. MLST was performed as described by Enright et al. [82]. Alleles and sequence types (STs) were assigned by submitting the DNA sequences to the MLST database (https://pubmlst.org/organisms/staphylococcus-aureus, accessed on 10 December 2021). *spa*-typing was performed via the amplification of the polymorphic region of the *Staphylococcus* protein A gene according to the previously described protocol and the obtained sequences were analyzed using the Ridom StaphType software (version 1.5, Ridom GmbH, Würzburg, Germany) [83]. *S. aureus* isolates were characterized by *agr*-typing using PCR for amplification of the *agr* genes (I-IV) using specific primers and conditions [84].

## 5. Conclusions

In this study, a moderate prevalence of staphylococci was isolated from owls admitted to a recovery center in Portugal. Nocturnal birds of prey may represent a reservoir of both CoNS and *S. aureus* presenting antimicrobial resistance determinants. A high diversity of *S. aureus* clonal lineages was identified, including one CC130 *mec*C-MRSA. Owls feed mostly on small mammals and insects, thus posing as vectors for transmission of pathogens. This may be the source of the staphylococci found among owls and the cause of the high diversity of staphylococci species and clonal lineages. Owls are in direct contact with many species of wild rodents, thus posing as vectors for the transmission of pathogens. Furthermore, antimicrobial resistance in wildlife may be a considerable hazard to human and animal health due to transmission through waterways and other environmental sources.

## Figures and Tables

**Table 1:** The distribution of CoNS and CoPS among the four owl species.

Owl Species	Number of Samples	Number of CoPS	Number of CoNS
*Tyto alba* (Barn owl)	41	7	9
*Bubo bubo* (Eurasian eagle-owl)	5	1	4
*Athene noctua* (Little owl)	25	8	8
*Strix aluco* (Tawny owl)	43	9	20
Total	114	25	41

**Table 2:** Genetic characterization and molecular typing of MRSA and MSSA isolates recovered from wild owls.

Isolate	Owl Species	Antimicrobial Resistance		Virulence Factors		Molecular Typing		
		Phenotype	Genotype	IEC Type	Other Genes	ST (CC)	*spa*	*agr*
VS2960	*Athene noctua*	PEN, FOX	*mec*C, *bla*Z-SCC*mec*XI		*hla*, *hlb*, *etd2*	1245 (130)	t843	III
VS2961	*Strix aluco*	PEN, FD			*hla*, *hlb*	49 (49)	t208	II
VS2962	*Strix aluco*	PEN	*bla*Z		*hla*, *hlb*	49 (49)	t208	II
VS2963	*Strix aluco*	Susceptible			*hla*, *hlb*	49 (49)	t208	II
VS2964	*Strix aluco*	Susceptible			*hla*, *hlb*	49 (49)	t9811	II
VS2965	*Athene noctua*	Susceptible			*hla*, *hlb*	49 (49)	t20169	II
VS2966	*Athene noctua*	Susceptible			*hla*, *hlb*	49 (49)	t208	II
VS2967	*Athene noctua*	Susceptible		E	*hla*	49 (49)	t208	II
VS2968	*Strix aluco*	PEN	*bla*Z	B	*hla*	8 (8)	t121	I
VS2969	*Athene noctua*	PEN, CIP	*bla*Z	B	*hla*	8 (8)	t121	I
VS2970	*Athene noctua*	PEN, FD	*bla*Z	B	*hla*	8 (8)	t121	I
VS2971	*Tyto alba*	Susceptible			*hla*, *hlb*	2328 (133)	t3750	III
VS2972	*Tyto alba*	PEN, FD			*hla*, *hlb*	2328 (133)	t3750	III
VS2973	*Tyto alba*	PEN, TET, FD	*tet*K		*hla*, *hlb*	2766 (121)	t12364	IV
VS2974	*Bubo bubo*	ERY	*erm*A	B	*hla*	718	t11333	II
VS2975	*Athene noctua*	PEN	*bla*Z		*hla*, *hlb*	30 (30)	t9413	III
VS2976	*Tyto alba*	TET	*tet*K		*hla*	692	t1422	I
VS2977	*Tyto alba*	Susceptible			*hla*, *hlb*	1956 (121)	t20223	IV
VS2978	*Strix aluco*	PEN, CIP, CD, FD	*bla*Z, *mph*C		*hla*, *hlb*	130 (130)	t843	III
VS2979	*Strix aluco*	Susceptible			*hla*, *hlb*	1640	t9853	IV
VS2980	*Athene noctua*	Susceptible			*hla*, *hlb*	6 (5)	t16615	I
VS2981	*Tyto alba*	Susceptible			*hla*, *hlb*	7184	t2247	I
VS2982	*Strix aluco*	Susceptible		E	*hla*	7352	t2143	I

PEN: penicillin; FOX: cefoxitin; FD: fusidic acid; CIP: ciprofloxacin; TET: tetracycline; ERY: erythromycin; CD: clindamycin; IEC: Immune Evasion Cluster; ST: sequence type; CC: clonal complex.

**Table 3:** Owl and staphylococci species identification and resistance genes identified.

Isolate	Staphylococci Species	Owl Species	Antimicrobial Resistance	
			Phenotype	Genotype
VS2985	*S. epidermidis*	*Bubo bubo*	PEN, FD	*bla*Z, *fus*B
VS2986	*S. epidermidis*	*Bubo bubo*	PEN, ERY, FD	*bla*Z, *msr*(A/B), *mph*C, *fus*B
VS2987	*S. sciuri*	*Strix aluco*	Susceptible	
VS2988	*S. sciuri*	*Tyto alba*	Susceptible	
VS2989	*S. sciuri*	*Tyto alba*	PEN	*mec*A
VS2990	*S. sciuri*	*Strix aluco*	PEN	*mec*A
VS2991	*S. sciuri*	*Tyto alba*	PEN, CD, TET, FD	*mec*A, *mph*C, *tet*K
VS2992	*S. sciuri*	*Tyto alba*	PEN, CD, FD	
VS2993	*S. sciuri*	*Tyto alba*	PEN, FOX, CD	*mec*A, *mph*C
VS2994	*S. sciuri*	*Strix aluco*	PEN	*mec*A
VS2995	*S. sciuri*	*Strix aluco*	PEN, CD, FD	
VS2996	*S. sciuri*	*Strix aluco*	Susceptible	
VS2997	*S. sciuri*	*Bubo bubo*	Susceptible	
VS2998	*S. sciuri*	*Strix aluco*	Susceptible	
VS2999	*S. sciuri*	*Strix aluco*	PEN, FD	
VS3000	*S. sciuri*	*Athene noctua*	PEN, FOX, CD, FD	*mec*A, *mph*C
VS3001	*S. sciuri*	*Athene noctua*	Susceptible	
VS3002	*S. sciuri*	*Strix aluco*	PEN	
VS3003	*S. sciuri*	*Strix aluco*	PEN, FD	*mec*A
VS3004	*S. sciuri*	*Athene noctua*	PEN, FOX, CD, TET, FD	*mec*A
VS3005	*S. sciuri*	*Athene noctua*	Susceptible	
VS3006	*S. sciuri*	*Tyto alba*	Susceptible	
VS3007	*S. lentus*	*Strix aluco*	CD	*mph*C
VS3008	*S. lentus*	*Strix aluco*	PEN, CD, TET, FD	*tet*K
VS3009	*S. lentus*	*Strix aluco*	PEN, CD, TET	*tet*K
VS3010	*S. lentus*	*Strix aluco*	CD, TET	*mph*C
VS3011	*S. lentus*	*Strix aluco*	Susceptible	
VS3012	*S. lentus*	*Strix aluco*	Susceptible	
VS3013	*S. lentus*	*Strix aluco*	TET	*tet*K
VS3014	*S. lentus*	*Tyto alba*	Susceptible	
VS3015	*S. lentus*	*Strix aluco*	PEN, CD, FD	*mec*A
VS3016	*S. lentus*	*Athene noctua*	FD	
VS3017	*S. lentus*	*Athene noctua*		*mec*A
VS3018	*S. vitulinus*	*Tyto alba*	Susceptible	*mec*A
VS3019	*S. vitulinus*	*Athene noctua*	PEN, FD	*mec*A
VS3020	*S. haemolyticus*	*Athene noctua*	Susceptible	
VS3021	*S. haemolyticus*	*Bubo bubo*	PEN, FOX, CIP, ERY, CD, TET, SXT	*mph*C, *msr*(A/B), *tet*O
VS3022	*S. saprophyticus*	*Tyto alba*	Susceptible	*mec*A
VS3023	*S. xylosus*	*Strix aluco*	PEN, FOX, ERY, CD, TET, C, FD	*mph*C, *tet*M, *tet*L
VS3024	*S. xylosus*	*Strix aluco*	ERY	
VS3025	*S. succinus*	*Strix aluco*	PEN	

C: chloramphenicol; CD: clindamycin; CIP: ciprofloxacin; ERY: erythromycin; FD, fusidic acid; FOX: cefoxitin; PEN: penicillin; SXT: trimethoprim-sulfamethoxazole; TET: tetracycline.

## Data Availability

Not applicable.

## Supplementary Materials

The following supporting information can be downloaded at: https://www.mdpi.com/article/10.3390/antibiotics11020240/s1, Table S1: Owl species, date of sample collection and distribution of the 66 staphylococci among owl samples.
