# Supplementary material for: Nocturnal Birds of Prey as Carriers of Staphylococcus aureus and Other Staphylococci: Diversity, Antimicrobial Resistance and Clonal Lineages
Source: Antibiotics (Basel). 2022 Feb 12;11(2):240. doi: 10.3390/antibiotics11020240 (PMC8868206; doi:10.3390/antibiotics11020240)
Supplement: Supplementary file 1 [file antibiotics-11-00240-s001.zip › antibiotics-1558508-supplementary.pdf]

Table S1. Owl species, date of sample collection and distribution of the 66 staphylococci among owl samples.

Scientific name	Common name	Date	Recovery center	Isolate recovered
Tyto alba	Barn owl	08-2018	PBG	*S. aureus* (VS2976)
Tyto alba	Barn owl	08-2018	PBG	*S. aureus* (VS2981)
Tyto alba	Barn owl	08-2018	PBG	*S. aureus* (VS2973), *S. sciuri* (VS2988)
Tyto alba	Barn owl	01-2019	PBG	*S. sciuri* (VS2989)
Tyto alba	Barn owl	03-2019	PBG	*S. pseudintermedius* (VS2983)
Tyto alba	Barn owl	03-2019	PBG	*S. aureus* (VS2977), *S. sciuri* (VS2991)
Tyto alba	Barn owl	06-2019	PBG	*S. sciuri* (VS2992)
Tyto alba	Barn owl	07-2019	PBG	
Tyto alba	Barn owl	11-2019	PBG	*S. sciuri* (VS2993)
Tyto alba	Barn owl	11-2019	PBG	
Tyto alba	Barn owl	01-2020	PBG	
Tyto alba	Barn owl	01-2020	PBG	
Tyto alba	Barn owl	01-2020	PBG	*S. sciuri* (VS3006)
Tyto alba	Barn owl	02-2020	PBG	
Tyto alba	Barn owl	05-2020	CERAS	
Tyto alba	Barn owl	05-2020	CERAS	
Tyto alba	Barn owl	05-2020	CERAS	
Tyto alba	Barn owl	05-2020	CERAS	
Tyto alba	Barn owl	05-2020	CERAS	*S. lentus* (VS3014)
Tyto alba	Barn owl	05-2020	CERAS	
Tyto alba	Barn owl	05-2020	CERAS	
Tyto alba	Barn owl	05-2020	CERAS	
Tyto alba	Barn owl	07-2020	CERAS	*S. vitulinus* (VS3018)
Tyto alba	Barn owl	09-2020	CERAS	
Tyto alba	Barn owl	06-2021	CERAS	
Tyto alba	Barn owl	06-2021	CERAS	
Tyto alba	Barn owl	06-2021	CERAS	*S. saprophyticus* (VS3022)
Tyto alba	Barn owl	06-2021	CERAS	
Tyto alba	Barn owl	06-2021	CERAS	
Tyto alba	Barn owl	06-2021	CERAS	
Tyto alba	Barn owl	06-2021	CERAS	
Tyto alba	Barn owl	06-2021	CERAS	
Tyto alba	Barn owl	06-2021	CERAS	
Tyto alba	Barn owl	06-2021	CERAS	
Tyto alba	Barn owl	05-2020	PBG	
Tyto alba	Barn owl	05-2020	PBG	
Tyto alba	Barn owl	06-2020	PBG	

Tyto alba	Barn owl	06-2020	PBG	*S. aureus* (VS2971)
Tyto alba	Barn owl	06-2020	PBG	*S. aureus* (VS2972)
Tyto alba	Barn owl	07-2020	PBG	
Tyto alba	Barn owl	07-2020	PBG	
Bubo bubo	Eurasian eagle-owl	07-2020	CERAS	*S. haemolyticus* (VS3020)
Bubo bubo	Eurasian eagle-owl	10-2020	CERAS	*S. epidermidis* (VS2986)
Bubo bubo	Eurasian eagle-owl	01-2021	CERAS	*S. sciuri* (VS2997)
Bubo bubo	Eurasian eagle-owl	01-2021	CERAS	
Bubo bubo	Eurasian eagle-owl	01-2021	CERAS	*S. aureus* (VS2974)
Athene noctua	Little owl	11-2018	PBG	*S. aureus* (VS2975)
Athene noctua	Little owl	01-2019	PBG	*S. sciuri* (VS3005)
Athene noctua	Little owl	02-2019	PBG	*S. aureus* (VS2969), *S. lentus* (VS3016)
Athene noctua	Little owl	05-2020	CERAS	*S. sciuri* (VS3004)
Athene noctua	Little owl	06-2020	CERAS	
Athene noctua	Little owl	06-2020	CERAS	
Athene noctua	Little owl	06-2020	CERAS	
Athene noctua	Little owl	06-2020	CERAS	*S. aureus* (VS2960)
Athene noctua	Little owl	06-2020	CERAS	
Athene noctua	Little owl	06-2020	CERAS	*S. aureus* (VS2965)
Athene noctua	Little owl	06-2020	CERAS	
Athene noctua	Little owl	06-2020	CERAS	
Athene noctua	Little owl	07-2020	CERAS	
Athene noctua	Little owl	07-2020	CERAS	
Athene noctua	Little owl	07-2020	CERAS	*S. aureus* (VS2966)
Athene noctua	Little owl	07-2020	CERAS	*S. sciuri* (VS3001)
Athene noctua	Little owl	10-2020	CERAS	*S. aureus* (VS2967), *S. lentus* (VS3017), *S. vitulinus* (VS3019), *S. haemolyticus* (VS3021)
Athene noctua	Little owl	05-2021	CERAS	*S. sciuri* (VS3000)
Athene noctua	Little owl	06-2021	CERAS	
Athene noctua	Little owl	06-2021	CERAS	*S. aureus* (VS2980)
Athene noctua	Little owl	06-2021	CERAS	
Athene noctua	Little owl	06-2021	CERAS	
Athene noctua	Little owl	06-2021	CERAS	
Athene noctua	Little owl	06-2021	CERAS	*S. aureus* (VS2970)
Athene noctua	Little owl	06-2021	CERAS	
Strix aluco	Tawny owl	06-2018	PBG	*S. aureus* (VS2961)
Strix aluco	Tawny owl	07-2018	PBG	*S. aureus* (VS2962)
Strix aluco	Tawny owl	03-2019	PBG	*S. sciuri* (VS2990)

Strix aluco	Tawny owl	03-2019	PBG	*S. pseuditermedius* (VS2984)
Strix aluco	Tawny owl	07-2019	PBG	*S. sciuri* (VS2987)
Strix aluco	Tawny owl	01-2020	PBG	
Strix aluco	Tawny owl	01-2020	PBG	*S. aureus* (VS2963)
Strix aluco	Tawny owl	05-2020	CERAS	
Strix aluco	Tawny owl	05-2020	CERAS	*S. lentus* (VS3008), *S. succinus* (VS3025)
Strix aluco	Tawny owl	05-2020	CERAS	*S. lentus* (VS3009), *S. xylosus* (VS3023)
Strix aluco	Tawny owl	05-2020	CERAS	*S. lentus* (VS3012)
Strix aluco	Tawny owl	05-2020	CERAS	*S. sciuri* (VS2998)
Strix aluco	Tawny owl	05-2020	CERAS	*S. sciuri* (VS2999), *S. lentus* (VS3015)
Strix aluco	Tawny owl	05-2020	CERAS	*S. sciuri* (VS2994)
Strix aluco	Tawny owl	05-2020	CERAS	*S. lentus* (VS3007)
Strix aluco	Tawny owl	05-2020	CERAS	*S. lentus* (VS3013)
Strix aluco	Tawny owl	05-2020	CERAS	*S. aureus* (VS2982)
Strix aluco	Tawny owl	05-2020	CERAS	
Strix aluco	Tawny owl	05-2020	CERAS	*S. lentus* (VS3011)
Strix aluco	Tawny owl	05-2020	CERAS	
Strix aluco	Tawny owl	05-2020	CERAS	
Strix aluco	Tawny owl	05-2020	CERAS	
Strix aluco	Tawny owl	06-2020	CERAS	*S. aureus* (VS2968)
Strix aluco	Tawny owl	09-2020	CERAS	
Strix aluco	Tawny owl	09-2020	CERAS	
Strix aluco	Tawny owl	09-2020	CERAS	*S. sciuri* (VS2995)
Strix aluco	Tawny owl	09-2020	CERAS	
Strix aluco	Tawny owl	10-2020	CERAS	*S. xylosus* (VS3024)
Strix aluco	Tawny owl	02-2021	CERAS	*S. aureus* (VS2978)
Strix aluco	Tawny owl	02-2021	CERAS	*S. sciuri* (VS2996)
Strix aluco	Tawny owl	03-2021	CERAS	*S. sciuri* (VS3002)
Strix aluco	Tawny owl	03-2021	CERAS	*S. aureus* (VS2975); *S. sciuri* (VS3003)
Strix aluco	Tawny owl	03-2021	CERAS	
Strix aluco	Tawny owl	04-2021	CERAS	*S. aureus* (VS2964)
Strix aluco	Tawny owl	04-2021	CERAS	
Strix aluco	Tawny owl	05-2021	CERAS	*S. aureus* (VS2979)
Strix aluco	Tawny owl	05-2021	CERAS	
Strix aluco	Tawny owl	05-2021	CERAS	
Strix aluco	Tawny owl	05-2021	CERAS	*S. lentus* (VS3010)
Strix aluco	Tawny owl	05-2021	CERAS	
Strix aluco	Tawny owl	07-2020	PBG	
Strix aluco	Tawny owl	06-2018	PBG	

Strix aluco	Tawny owl	06-2018	PBG

PBG: Parque Biológico de Gaia, CERAS: Centro de Estudos e Recuperação de Animais Selvagens
